# Dietary patterns in North and South India: a comparison with EAT-Lancet dietary recommendations

**DOI:** 10.1111/jhn.13222

**Published:** 2023-08-09

**Authors:** Anjali Ganpule-Rao, Manisha Dubey, Himanshi Pandey, Rosemary Green, Kerry Ann Brown, Nikhil Srinivasapura Venkateshmurthy, Prashant Jarhyan, Avinav Prasad Maddury, Rajesh Khatkar, Dorairaj Prabhakaran, Sailesh Mohan

**Affiliations:** 1 Centre for Chronic Disease Control, New Delhi, India; 2 London School of Hygiene and Tropical Medicine, UK; 3 University of Exeter, UK; 4 Public Health Foundation of India, New Delhi, India; 5 Deakin University, Melbourne Australia

**Keywords:** Indian diets, EAT-Lancet recommendations, adults

## Abstract

**Background:**

Environmentally sustainable diets are represented in the EAT-Lancet recommendations developed by the Lancet Commission on Planetary Health in 2019. Very few studies have compared Indian diets with the EAT-Lancet recommendations, and we did this using primary dietary consumption data from adults in north and south India.

**Methods:**

Data from 8762 adults (52.4±11.7 years) residing in Sonipat and Vizag India were collected on socio-demographic characteristics, wealth index (household assets), and dietary intake (food frequency questionnaire: 9 food groups). The quantity consumed and energy from each food group was compared with the EAT-Lancet recommendations. We studied the likelihood of deficit or excess in consumption compared to the EAT-Lancet recommendations by different socio-demographic factors.

**Results:**

Half of the participants were women and half resided in rural areas. Vegetables and fruits were consumed in lower quantity, while dairy and added fats were consumed in higher quantity than recommended by the EAT-Lancet recommendations. For whole grains, female gender and poorest wealth index were the factors associated with deficit or no consumption, while for vegetables and fruits, it was poorest wealth index and residence in rural areas (p<0.05, all). Rural residence poor, and poorest wealth index were associated with excess consumption of dairy and added fats (p<0.05, all).

**Conclusions:**

The diets of the study participants were mainly plant-based, high in dairy but lacking in nutrient-rich foods like vegetables and fruits. Appropriate policy actions for making healthy sustainable diets and micronutrient-rich foods available and affordable to all with a particular focus on the poor and rural populations are warranted.

## Introduction

Providing the growing global population with healthy diets from sustainable food systems is an immediate challenge. In 2010, the Food and Agriculture Organization (FAO) defined sustainable and healthy diets as those with “low environmental impacts that contribute to healthy life for present and future generations and are protective and respectful of biodiversity and ecosystems, culturally acceptable, accessible, economically fair and affordable; nutritionally adequate, safe and healthy” ^(1)^. Considering these, the Sustainable Development Goals (SDGs) were set that promise to ensure food security and nutrition within sustainable food systems ^(2)^. However, the current global trends of diets and the food systems that produces those diets, are not aligned with the above goals as they are neither healthy nor sustainable ^(3,4)^. This has implications for achieving the SDGs by 2030. It is acknowledged globally the disease burden due to dietary risks is alarmingly high and we are unlikely to universally meet the SDG 2 goal of Zero Hunger ^(3,4)^.

In this regard, large-scale and coordinated efforts are required to transform the global food systems and achieve the SDG goals. Efforts are required to make food systems environmentally sustainable and toward this understanding the impact of different foods and their environmental impacts is essential ^(5)^. For example, animal-sourced foods tend to have higher environmental impacts than plant-based foods, and within a food group there can be considerable variation dependent on the environmental indices used (greenhouse gas emissions, water use etc.), how, where, and when a food was produced, and its level of processing ^(6)^. The EAT-Lancet recommendations considered the environmental and health impacts of foods and set universal scientific targets for global diets, to make food systems healthy (safe and nutritious) and environmentally sustainable (operate within planetary boundaries) ^(7)^. It provides quantitative scientific targets or ranges for different food groups that will enable 10 billion people to consume a healthy diet within safe planetary boundaries by 2050, underlining that shifts in dietary patterns around the world are both possible (in theory) and necessary. The EAT-Lancet diet includes reference ranges and absolute amounts, in grams per day and kcal per day, for nine food groups: whole grains, tubers and starchy vegetables, fruits, other vegetables, dairy foods, protein sources, added fats, and added sugars. These recommendations provide a way to compare the health and sustainability of diets around the world. For example, the EAT-Lancet diet has been associated with a lower risk of ischaemic heart disease and diabetes ^(8)^ and overall mortality ^(9)^. Meeting the dietary recommendations has also been related to achieving higher micronutrient adequacy of diets in rural women of reproductive age from LMICs ^(10)^.

India needs a greater push to achieve SDG 2 zero hunger goal as ensuring universal food as well as nutrition security remains a challenge ^(11, 12)^. The burden of diet related non-communicable diseases is also increasing following “nutrition transitions,” as dietary patterns change to those observed in many high-income countries ^(13)^. In this context, few studies have compared Indian diets against the EAT-Lancet recommendations. For example, a study using the household Consumption Expenditure Survey (CES) conducted by the National Sample Survey Organization (NSSO) of India in 2011–12, showed that Indian food expenditures for different food groups were much lower than the EAT-Lancet recommendations ^(14)^. Another study using primary data on food prices and household food purchases, and secondary data on food expenditures for a period of 12 months in 2018–19 indicated similar results ^(15)^. However, these studies, were not based on primary consumption data which is essential to make the comparison. The current analysis addresses this gap by using primary consumption data and it can inform context-specific targeted actions. We provide a comparison using primary data collected from two diverse Indian populations, which include adults from both rural and urban areas and across different socio-economic strata. This study aimed to identify the dietary gaps in quantity and calorie consumption in Indian diets as compared with EAT-Lancet recommended diets and investigate the differences by region (north or south), place of residence (rural or urban), gender and socioeconomic status.

## Methods

### Study design

The analysis presented in this paper is based on data from the UDAY cohort study’s baseline follow-up survey conducted during October 2018-February 2019 among 9005 adults aged ≥ 30 years from urban and rural households in Sonipat (north India) and Vizag (south India). The UDAY cohort’s methodology has been published elsewhere ^(16)^. Briefly, for the baseline UDAY study the participants were selected using multistage cluster random sampling and participants from the same primary sampling unit were followed up through a cross-sectional survey for the baseline follow-up survey after 5 years.

### Ethics

Ethics approval was obtained from the Institutional Ethics Committee (IEC) of the Public Health Foundation of India (IRB No: IRB00006330). Participants willing to participate and who provided informed written consent were included in the study.

### Measurements

All the measurements were carried out by trained field staff by using standardised methodology, entered in a computer-assisted personal interview (CAPI) platform, and were closely supervised and evaluated by the research staff for quality control.

### Demographics

Information on residence (urban or rural), site and state (Sonipat, Haryana or Vizag, Andhra Pradesh), age, gender, and employment status were collected through a questionnaire.

### Wealth index

The wealth index was calculated based on a paper authored by Filmer et al ^(17)^. Using a questionnaire, information on household assets (radio, television, computer, etc.) and facilities (water supply, toilet, electricity, etc.) were collected. The wealth index was constructed using these indices separately for rural and urban areas. Those belonging to the first quintile were defined as the poorest and those in the fifth defined as the richest.

### Dietary food groups

In this study, the dietary intakes were measured using a food frequency questionnaire containing 23 groups. For the EAT-Lancet comparison, the food groups were merged into the nine EAT Lancet food groups according to their nutritional content and additional one refined group as shown in Table 1. As half the participants were vegetarian (consumed no egg, meat, or fish), we divided the protein group into vegetarian and non-vegetarian categories. Additionally, we included white rice and refined flours under the refined category as the tenth group.

### Quantity (grams/day) and energy (kcal/day) calculation

In the UDAY questionnaire the participants were initially asked to report the consumption of various foods in four frequency categories: daily, weekly, monthly, and rarely (never or less than once a month). Further, they were asked about the portion size of servings per day or the amount eaten each sitting, using standardized food models (bowls, cups, spoons, etc.). Using the above information and standardized recipe manual ^(18)^ the daily raw quantity of various foods consumed by the participants was calculated. Based on the quantity of all food groups (grams/day) (Table 1) the daily energy consumed was calculated using the reference values for raw food from the Indian Food Composition Table ^(19)^ and for cooked food from Nutritive values of some Indian food preparations ^(20)^.

### Statistical analysis

Continuous variables were summarised as means and categorical variables as frequencies. We compared the daily quantity and energy consumption of the participants with the EAT-Lancet recommendations. The EAT-Lancet pattern provides ranges in addition to single values for most of its recommendations to account for uncertainty and accommodate diverse eating patterns according to individual preferences and cultural contexts. The means from the UDAY diet patterns were compared with the ranges or single values as provided by EAT-Lancet recommendations. We also compared the diets by residence (urban and rural) and states (north and south). Further, using multivariable logistic regression models at 95% CI, the likelihood of consuming lower than the recommended intake (none or deficit) for whole grains, vegetables and fruits and the likelihood of consuming higher than the recommended (excess) intake for dairy and added fats by gender, wealth index categories and rural-urban residence, controlling for age were studied. The statistical analysis was completed using Stata 16.1 (Stata Corp)/ SPSS 22 (IBM, India).

## Results

Consumption data was available for 8762 (97.3%) out of 9005 recruited participants (Supplementary table 1). Of these participants, 50% were women and resided in rural areas. The mean age of the participants was 52.4 (±SD11.7) years.

### Proportion of participants following EAT-Lancet recommendations

More than 80% of the participants consumed all nine EAT-Lancet recommended food groups, except for the non-vegetarian group. Half of the study participants, specifically those residing in Sonipat, were identified as vegetarians (no consumption of eggs, meat, and fish) (Table 2).

### Comparison of quantity as compared to EAT-Lancet

When we compared the quantity of the food groups consumed, except for dairy, vegetables, and fruits, all EAT Lancet food groups were consumed within the target ranges but lower than average value suggested by the EAT-Lancet recommendations (Table 2). The dairy consumption was above the recommended single value of 250 g/d, although within the upper bound of the range of 0-500 g/d). Vegetable and fruit consumption were substantially less, on average, than the lower boundary of the EAT-Lancet range.

When compared by region, the mean quantity consumed of whole grains, all vegetables, fruits, dairy, and added fats was higher in Vizag than in Sonipat (Supplementary Table 2). However, the mean quantity consumed of starchy vegetables and added sugars were higher in Sonipat than in Vizag (p <0.01 for all). When compared by urban/ rural residence, consumption of starchy vegetables, all vegetables, fruits, and protein-rich foods were higher in urban areas. Whereas, the consumption of whole grains and dairy products was higher in rural areas (p <0.01 for all). Consumption of starchy vegetables and all vegetables were higher among women, while consumption of non-vegetarian protein sources was higher among men. Consumption of whole grains and added fats were higher among participants belonging to the poorest wealth index category, while consumption of vegetables, fruits, and dairy was highest among participants belonging to the richest wealth index.

### Comparison of energy

Corresponding to comparisons in quantity, additional information was identified by looking at the contribution of each food group to daily energy (kcal) intake (Figure 2). Dairy and added fats contributed remarkably higher energy to daily diets than recommended by the EAT-Lancet diet. On the other hand, fruits (80%) and protein sources (83%) contributed remarkably lower energy than recommended by EAT-Lancet diet. The average energy intake of the participants was 1560 kcal/day. Participants from Vizag consumed average 1925 kcal/day, while participants from Sonipat consumed average 1261 kcal/day. Only 0.5% of the participants consumed ≥2500kcal/day and 10% consumed >2000 kcal/day as recommended by EAT-Lancet diet (results not shown in tables).

### Comparison with EAT-Lancet recommended ranges

**Figure 1:**
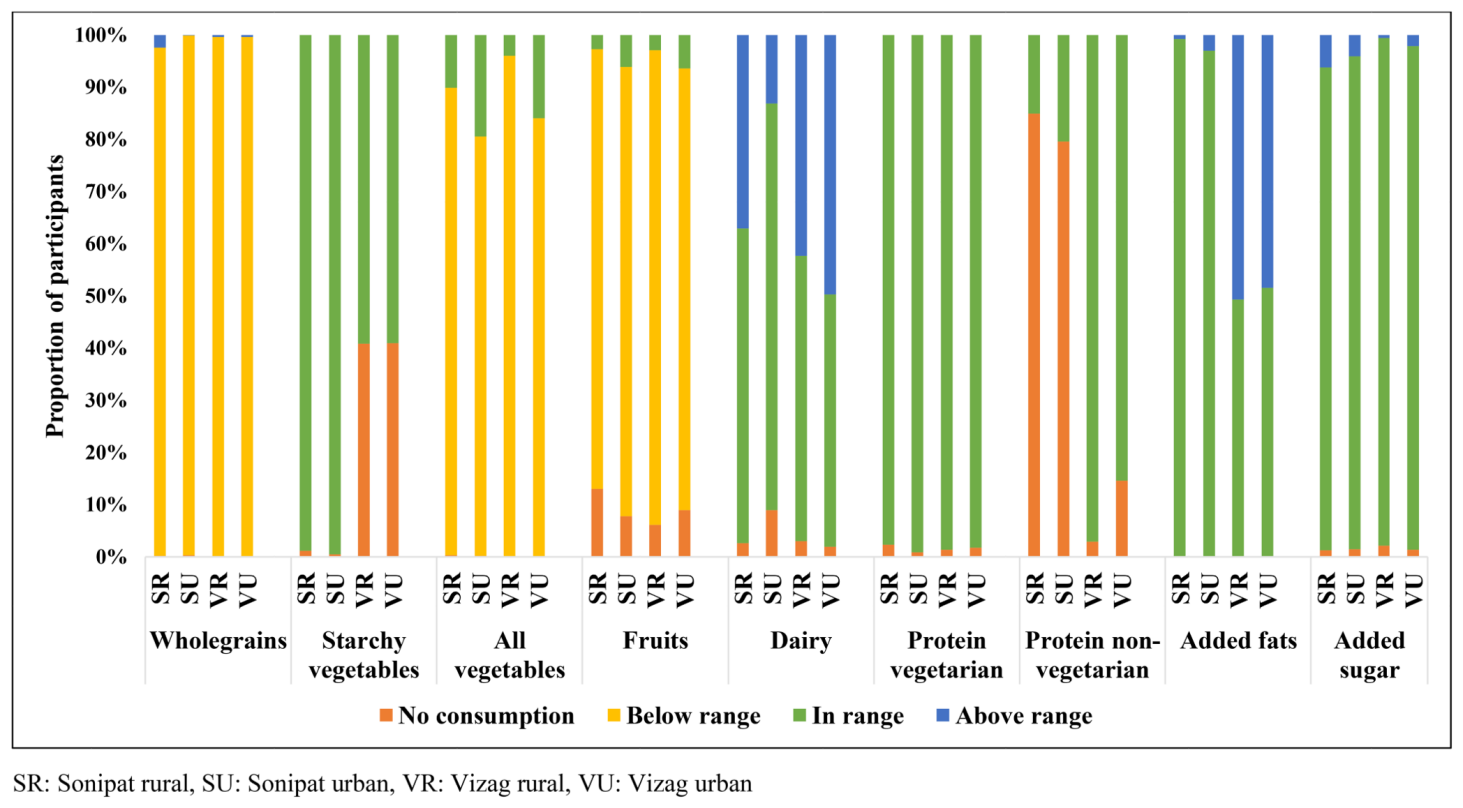
Consumption patterns (no, below, within or above the EAT-Lancet recommendation) of various food groups by the study participants

**Figure 2:**
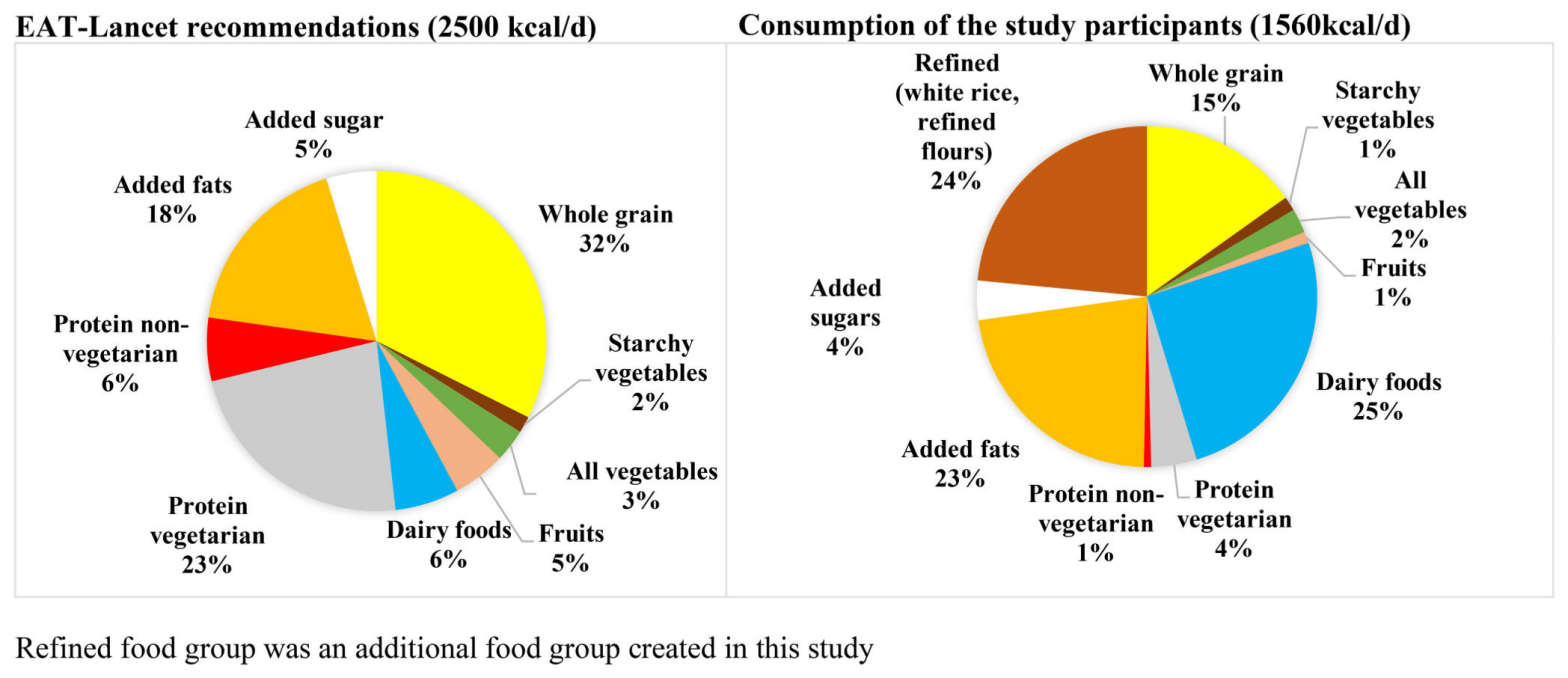
Daily calorie consumption of the study participants compared with EAT-Lancet recommendations

Using the ranges suggested by EAT-Lancet recommendations, we classified the participants from the two states and, urban and rural places of residence into no consumption, below, in and above range categories (Figure 1). For starchy vegetables, dairy, protein sources (vegetarian and non-vegetarian), added fats, and sugar there are no lower-range recommendations provided by EAT-Lancet diet. Thus, we could not classify participants into a below-range category. Most of the participants belonged to the deficit or no consumption categories for whole grains, vegetables, and fruits. Participants from Sonipat did not consume non-vegetarian protein sources, while dairy was consumed in the above range mainly by participants from rural Sonipat and Vizag (both urban and rural). Added fats were consumed in the above range mainly by participants from Vizag.

### Socio-demographic factors associated with deficit or excess consumption of different EAT-Lancet groups

We studied socio-demographic factors like age, gender, wealth index and residence that may be related to the likelihood (Table 3) of belonging to the deficit or no consumption groups relating to foods that were least consumed, i.e., whole grains, all vegetables, and fruits (as shown in Figure 1). For whole grains, female participants (OR 2.69 95% CI 1.67, 4.33) and those belonging to the poorest wealth index (OR 3.50 95% CI: 1.50, 8.16) had a higher likelihood to fall into the deficit or no consumption category than their counterparts. For all vegetables and fruits, those belonging to the poorest wealth index (Vegetables: OR 4.49 95% CI: 3.53, 5.70; Fruits: OR 6.01 95% CI: 4.03, 8.94) and those residing in rural areas (Vegetables: OR 2.94 95% CI: 2.55, 3.38; Fruits: OR 2.30 95% CI: 1.86, 2.86) had a higher likelihood to fall into the deficit or no consumption category than their counterparts (p<0.05, all). For all three food groups increasing age was associated with the higher likelihood of participants falling into the deficit or no consumption category.

Dairy and added fats were two food groups consumed in higher quantity than recommended by some of the participants (Table 4). For dairy (OR 1.60 95% CI: 1.47, 1.76); and for added fats, (OR 1.20 95% CI: 1.08, 1.33) those residing in rural areas had a higher likelihood of falling into the excess consumption category than their counterparts (p<0.05, all).

## Discussion

This study compares food consumption with the EAT-Lancet recommendations among adult men and women from North and South India and found that Indian diets currently are inadequate in terms of quantity for many food groups compared to Eat-Lancet recommendations. Quantities consumed were low for cereals, vegetables, and fruits. Correspondingly, there were substantial differences in the contribution of each food group to the daily energy intake than recommended. Those contributed by dairy and added fats were very high and that of cereals, vegetables, fruits, and protein sources were notably lower than recommended by EAT-Lancet diet. There were additional contributions by the refined food group. Place of residence, wealth index, and gender were the major factors influencing deficit in the consumption patterns compared to the EAT-Lancet recommendations. Notably, those residing in rural areas and belonging to the poor wealth index category were found to have the most inadequate diets, as they were deficient in nutrient-rich foods like vegetables and fruits.

Our paper presents results in the context of the first strategy outlined in the EAT-Lancet recommendations, which aims to secure international and national commitments for transitioning towards healthy diets ^(21)^. The scientific targets set out by this Lancet Commission provide guidance about the necessary shift, which consists of high consumption of plant-based foods and substantially limiting animal-source foods. However, these recommendations may not be directly applicable to India as the dietary patterns are at considerable variance from the EAT-Lancet recommendations. A study analysing national data from the food expenditure survey conducted a decade back reported that Indian diets have shifted away from cereals to higher consumption of dairy ^(14, 22)^. These trends appear to have continued as in the current study, consumption of cereals and legumes was low, while dairy was the prominent food group consumed in adequate or excess quantities in both rural and urban settings as well as in north and south India. This is disconcerting as dairy has a higher carbon footprint and thus can have higher adverse environmental impacts. Apart from dairy, added fat consumption, especially among the participants belonging to the poorest wealth index category was observed. As reported by a previous study in urban slums, fat food consumption was higher in low socioeconomic neighbourhoods ^(23)^. As high-fat diets are known to be closely associated with metabolic risks ^(24)^ and cardiometabolic risks are rising in low socioeconomic groups, added fat consumption needs to be reduced. Our findings are consistent with previous research indicating that Indian diets often lack essential fruits and vegetables ^(14)^, which are critical for addressing widespread micronutrient deficiencies in Vitamin A, C, and iron ^(22)^, as well as preventing non-communicable diseases such as diabetes and hypertension ^(25)^.

Based on our findings the consumption of protein foods from nonvegetarian sources is lower than EAT-Lancet recommendations. These findings are supported by previous surveys by the Government of India ^(26)^ and a report by the United Nations Food and Agriculture Organization ^(27)^. Thus, Indians consume less cereals, proteins (vegetarian as well as non-vegetarian foods), vegetables, and fruits, and have high dairy and high-fat intake. While giving suggestions to maintain a balance between health and sustainability, the above consumption patterns of Indians need to be considered.

The focus of EAT-Lancet diet is on the environmental sustainability of food production and the health consequences of final consumption. A transformation of the global food system must ultimately involve multiple stakeholders, from individual consumers to policymakers and actors along the food value chain, working together toward the shared global goal of healthy and sustainable diets for all. In this regard in India, apart from improving consumer’s knowledge regarding environmentally sustainable consumption and reducing food wastage at the household level, other stakeholders of the food value chain also must be addressed. For example, there is a need for policies to address post-harvest losses that are 30-40% for vegetables and fruits (28, 29). This will help to address the issue of accessibility, availability, and affordability of fruits and vegetables, consumption of which was particularly lower among those belonging to poor wealth index and residing in rural areas.

While computing and comparing our diets according to the EAT-Lancet recommendations, we faced certain limitations. Considering the socio-cultural and regional differences worldwide, the lower range for certain food groups like whole grains, starchy vegetables, and protein groups is zero for EAT-Lancet, hence we could not estimate the gaps in diets for these foods. Considering that the global population consumes both vegetarian and non-vegetarian sources of protein, the EAT-Lancet diet has given combined recommendations for protein sources. However, half of the study population from Sonipat did not consume non-vegetarian protein sources. Hence, we had to separate out the vegetarian and non-vegetarian protein sources in our results. The EAT-Lancet recommendations also did not specify the type of food under each category. For example, the type of rice (brown or polished), which whole grains can be considered, etc. So, we had to compute the groups according to our understanding. Thus, it may be useful for the EAT-Lancet recommendations to consider providing separate recommendations for micronutrient-rich foods, considering the inadequate dietary patterns in LMICs. Also, high sugar, salt, and oil/fat-containing snacks and processed foods are now part of everyday diets in India and contributed ~15% of daily energy as shown in our recent analysis of snack consumption in the same population ^(24)^. Considering the heavy public health burden of diet-related cardio-metabolic conditions in India and other LMICs such recommendations are of tremendous importance for advancing prevention and control of these conditions.

Though the total calorie recommended by EAT-Lancet diet and the ICMR is similar (up to 2500kcal), the contributions recommended for different food groups vary considerably. The EAT-Lancet diet recommends lower whole grains, vegetables, and dairy foods than the ICMR ^(30)^ recommendations. Thus, while suggesting sustainable and healthy diets for India and implementing them at the national level, the EAT-Lancet recommendations must be adapted considering the ICMR and EAT Right of the Food Safety and Standards Authority of India (FSSAI) guidelines as a reference point. Of note, the EAT Right guidelines aims to transform the country’s food system in order to ensure safe, healthy, and sustainable food for all Indians. It includes promotion of sustainable eating habits, such as promoting local and seasonal foods, limiting unhealthy food choices, and conduct of mass awareness activities (^31^).

## Conclusions

The diets of the study participants were mainly plant-based, high in dairy but lacking in nutrient-rich foods like fruits and vegetables, and were at variance from the EAT-Lancet recommendations. The study indicates the need for appropriate policy actions for making healthy sustainable diets and micronutrient-rich foods available and affordable to all with a particular focus on the poor and rural populations. Unlike industrialized countries, consumption of red meat and other non-vegetarian foods, high in carbon footprint. is not a major issue. However, a shift towards higher dairy consumption can lead to high carbon footprint from Indian diets.

## Supplementary Material

Supplementary table 1

Supplementary table 2

## Figures and Tables

**Table 1:** Distribution of EAT-Lancet and UDAY food groups

Serial No.	EAT-Lancet food groups	UDAY food groups
1	Whole grains	Rice, whole wheat, refined flour
2	Starchy vegetables	Starchy vegetables
3	All vegetables	Green leafy vegetables, other vegetables, uncooked raw vegetables
4	Fruits	Sweet fruits, other fruits
5	Dairy foods	Milk, milk products
6	Protein vegetarian: Legumes, nuts, soy food, peanuts	Legumes
7	Protein non-vegetarian: Chicken, other poultry, eggs, fish, beef, lamb, and pork	Poultry, eggs, fish, shell fish, meat, trimmed meat, organ meats
8	Added fats	Deep fried (2 groups), Desserts (2 groups) Calculated from mixed food groups: daily foods plus fried foods plus desserts
9	Added sugar	
10	Additional	Refined flour and white rice

**Table 2:** Average raw median quantity of different foods consumed by the participants

Eat-Lancet food groups	Recommended quantity, g/day	% Consumers (N=8762)	Daily quantity consumed (g) Median (IQR)
**Whole grains**	232	99.8	103.0 (59.3, 210.0)
**Starchy vegetables**	50 (0-100)	79.9	32.0 (10.7, 53.3)
**All vegetables**	300 (200-600)	99.8	101.3 (64.0, 160.0)
**Fruits**	200 (100-300)	91.0	28.0 (14.0, 56.0)
**Dairy foods**	250 (0-500)	95.9	400.0 (280.0, 600.0)
**Total protein**	209 (0-197)	99.2	25.5 (16.0, 40.0)
**Protein vegetarian**	125 (0-175)	98.4	16.0 (8.0, 32.0)
**Protein non-** **vegetarian**	84 (0-197)	45.5	4.0 (0.0, 15.0)
**Added fats**	52(0-80)	100.0	37.4 (25.0, 51.8)
**Added sugar**	31(0-31)	98.4	13.3 (12.0, 18.1)

Grey highlighted rows shows significantly lower consumption

**Table 3:** Likelihood of having deficit consumption by socio-demographic characteristics

Sociodemographic factors	Categories	Exp(B) Adjusted for all factors	95% CI for Exp(B)		p value
			Lower Bound	Upper Bound	
**Whole grains**					
Age		1.03	1.01	1.05	0.01
Gender (Male as reference)	Female	**2.69**	**1.67**	**4.33**	**0.00**
Wealth index (Richest as reference)	1 Poorest	**3.50**	**1.50**	**8.16**	**0.00**
	2	3.00	1.38	6.50	0.01
	3	1.69	0.89	3.20	0.11
	4 Rich	1.29	0.71	2.35	0.41
Residence (Urban as reference)	Rural	0.16	0.08	0.32	0.00
**All vegetables**					
Age		**1.01**	**1.01**	**1.02**	**0.00**
Gender (Male as reference)	Female	**1.31**	**1.14**	**1.50**	**0.00**
Wealth index (Richest as reference)	1 Poorest	**4.49**	**3.53**	**5.70**	**0.00**
	2	4.22	3.34	5.32	0.00
	3	2.14	1.77	2.59	0.00
	4 Rich	1.49	1.24	1.78	0.00
Residence (Urban as reference)	Rural	**2.94**	**2.55**	**3.38**	**0.00**
**Fruits**					
Age		**1.01**	**1.00**	**1.02**	**0.00**
Gender (Male as reference)	Female	1.11	0.90	1.37	0.32
Wealth index (Richest as reference)	1 Poorest	**6.00**	**4.03**	**8.94**	**0.00**
	2	**4.26**	**3.01**	**6.03**	**0.00**
	3	3.25	2.39	4.41	0.00
	4 Rich	2.14	1.62	2.81	0.00
Residence (Urban as reference)	Rural	**2.30**	**1.85**	**2.86**	**0.00**

**p< 0.01**

**Table 4:** Likelihood of having excess consumption by socio-demographic characteristics

Socio demographic factors	Categories	Exp(B) Adjusted for all factors	95% CI for Exp(B)		p value
			Lower Bound	Upper Bound	
**Dairy**					
Age		1.00	1.00	1.00	0.77
Gender (Male as reference)	Female	0.94	0.86	1.03	0.17
Wealth index (Richest as reference)	1 Poorest	0.72	0.62	0.83	0.00
	2	**1.22**	**1.06**	**1.40**	**0.01**
	3	1.26	1.09	1.44	0.00
	4 Rich	1.09	0.95	1.25	0.23
Residence (Urban as reference)	Rural	**1.60**	**1.47**	**1.76**	**0.00**
**Added fats**					
Age		0.99	0.98	0.99	0.00
Gender (Male as reference)	Female	0.92	0.83	1.02	0.10
Wealth index (Richest as reference)	1 Poorest	**5.25**	**4.30**	**6.41**	**0.00**
	2	**6.70**	**5.49**	**8.16**	**0.00**
	3	4.45	3.65	5.44	0.00
	4 Rich	2.37	1.92	2.93	0.00
Residence (Urban as reference)	Rural	**1.20**	**1.08**	**1.33**	**0.00**

**p< 0.01**

## Highlights

- Compared with the EAT-Lancet recommendations, Indian diet were deficit in fruits and vegetables consumption but exceeded in dairy and added fats.
- To ensure sustainable and healthy diets in India, the EAT-Lancet recommendations should be adopted considering ICMR guidelines.
- Female gender, rural residence and poorest wealth index were associated with lower nutrient rich foods consumption.
- Urgent policy actions are needed to make healthy and sustainable diets locally available accessible and affordable to all
