# Supplementary material for: Dietary patterns in North and South India: a comparison with EAT-Lancet dietary recommendations
Source: J Hum Nutr Diet. Author manuscript; Available in PMC 2024 Jul 24. (PMC7616295; doi:10.1111/jhn.13222)
Supplement: Supplementary table 1 [file EMS197578-supplement-Supplementary_table_1.docx]

**Supplementary Table 1: Socio-demographic characteristics of the study participants**

| **Sample characteristics** | **Categories** | **Participants**  N (%) |
| --- | --- | --- |
| **State** | Sonipat | 4558(52.0) |
|  | Vizag | 4204(48.0) |
| **Residence** | Rural | 4915(56.1) |
|  | Urban | 3847(43.9) |
| **Age** | 30-44 years | 2514(28.7) |
|  | 45-59 years | 3760(42.9) |
|  | ≥60 years | 2488(28.4) |
| **Gender** | Male | 3813(43.5) |
|  | Female | 4949(56.5) |
| **Wealth Index** | Poorest | 1697(19.4) |
|  | Poor | 1732(19.8) |
|  | Middle | 1832(20.9) |
|  | Rich | 1745(19.9) |
|  | Richest | 1756(20.0) |
| **Employment** | Un-employed | 93(1.1) |
|  | Housewife | 3409(38.9) |
|  | Retired | 785(9.0) |
|  | Employed | 4473(51.1) |
