# Supplementary material for: Dietary patterns in North and South India: a comparison with EAT-Lancet dietary recommendations
Source: J Hum Nutr Diet. Author manuscript; Available in PMC 2024 Jul 24. (PMC7616295; doi:10.1111/jhn.13222)
Supplement: Supplementary table 2 [file EMS197578-supplement-Supplementary_table_2.docx]

**Supplementary Table 2: Raw quantity (Mean ± SD) of various food groups consumed by the participants by sample characteristics**

| **Food groups** | **Whole**  **grains** | **Starchy vegetables** | **All vegetables** | **Fruits** | **Dairy** | **Protein vegetarian** | **Protein non-vegetarian** | **Added fats** | **Added sugar** |
| --- | --- | --- | --- | --- | --- | --- | --- | --- | --- |
| **State** | | | | | | | | | |
| Sonipat (n=4558) | 74.2±39.4 | 48.4±27.3 | 112.4±81.0 | 37.1±31.0 | 410.7±253.6 | 19.9±13.9 | 2.2±7.5 | 26.8±9.5 | 16.5±8.1 |
| Vizag (n=4204) | 176.8±45.2 | 14.1±11.3 | 123.8±56.7 | 40.1±32.0 | 505.8±219.3 | 21.8±14.5 | 16.5±11.8 | 52.2±11.5 | 13.0±5.9 |
| p value | <0.001 | <0.001 | <0.001 | <0.001 | <0.0001 | <0.001 | <0.001 | <0.001 | <0.001 |
| **Residence** | | | | | | | | | |
| Rural (n=4915) | 139.3±74.0 | 34.7±27.7 | 106.9±60.3 | 35.0±29.4 | 496.7±243.3 | 16.8±11.0 | 8.5±10.1 | 39.3±16.2 | 14.6±7.4 |
| Urban (n=3847) | 103.3±48.4 | 38.1±28.8 | 131.9±79.9 | 43.0±33.5 | 408.2±232.1 | 25.9±16.2 | 9.9±14.2 | 38.6±16.8 | 15.1±7.3 |
| p value | <0.001 | <0.001 | <0.001 | <0.001 | <0.001 | <0.001 | <0.001 | 0.05 | <0.001 |
| **Gender** | | | | | | | | | |
| Male (n=3813) | 124.8±67.8 | 33.3±25.9 | 122.0±73.1 | 38.7±31.4 | 462.3±248.5 | 20.5±13.8 | 10.1±12.6 | 39.0±16.6 | 14.5±7.6 |
| Female (n=4949) | 122.5±65.4 | 38.5±29.7 | 144.6±68.5 | 38.4±31.6 | 455.7±237.7 | 21.0±14.6 | 8.3±11.6 | 39.0±16.3 | 15.1±7.2 |
| p value | 0.10 | <0.001 | <0.001 | 0.65 | 0.22 | 0.10 | <0.001 | 0.99 | <0.001 |
| **Wealth index** | | | | | | | | | |
| Poorest (n=1697) | 153.5±66.4 | 31.1±27.8 | 102.7±56.5 | 28.8±26.3 | 412.5±237.4 | 18.6±12.3 | 12.1±11.4 | 43.8±16.2 | 13.2±6.4 |
| Poor (n=1732) | 149.5±63.5 | 28.9±27.0 | 111.3±55.7 | 34.5±29.1 | 473.9±235.3 | 20.9±13.5 | 13.4±12.6 | 45.2±15.8 | 13.8±6.7 |
| Middle (n=1832) | 127.8±64.8 | 34.3±27.4 | 117.0±68.7 | 36.8±29.9 | 473.9±241.0 | 21.1±14.8 | 10.2±12.2 | 40.8±16.7 | 14.6±7.4 |
| Rich (n=1745) | 104.1±59.7 | 39.9±29.3 | 123.2±74.0 | 41.2±31.7 | 467.5±247.7 | 21.6±15.2 | 6.4±11.8 | 35.2±15.5 | 15.8±7.6 |
| Richest (n=1756) | 83.7±48.6 | 43.6±26.8 | 134.5±88.5 | 49.8±35.3 | 461.0±245.3 | 21.7±14.9 | 3.5±9.5 | 30.1±12.7 | 16.6±8.1 |
| p value | <0.001 | <0.001 | <0.001 | <0.001 | <0.001 | <0.001 | <0.001 | <0.001 | <0.001 |
